# Thromboembolic and infectious complication risks in TKA and UKA: evidence from a Japanese nationwide cohort

**DOI:** 10.1186/s43019-025-00273-6

**Published:** 2025-05-08

**Authors:** Yu Mori, Kunio Tarasawa, Hidetatsu Tanaka, Masayuki Kamimura, Kento Harada, Naoko Mori, Kiyohide Fushimi, Toshimi Aizawa, Kenji Fujimori

**Affiliations:** 1 Department of Orthopaedic Surgery, Tohoku University Graduate School of Medicine, 1-1 Seiryo-Machi, Aoba-Ku, Sendai, Miyagi 980-8574 Japan; 2 Department of Health Administration and Policy, Tohoku University Graduate School of Medicine, 2-1 Seiryo-Machi, Aoba-Ku, Sendai, Miyagi 980-8574 Japan; 3 Department of Radiology, Akita University Graduate School of Medicine, 1-1-1 Hondo, Akita, 010-8543 Japan; 4 Department of Health Policy and Informatics, Tokyo Medical and Dental University Graduate School of Medicine and Dental Sciences, 1-5-45 Yushima, Bunkyo-Ku, Tokyo, 113-8519 Japan

**Keywords:** Total knee arthroplasty, Unicompartmental knee arthroplasty, Deep vein thrombosis, Pulmonary embolism, Surgical site infection, Nationwide database

## Abstract

**Introduction:**

Total knee arthroplasty (TKA) and unicompartmental knee arthroplasty (UKA) are widely used to treat knee osteoarthritis. TKA significantly contributes to long-term pain relief and joint function improvement, while UKA offers faster recovery and reduced early complications. However, TKA and UKA complication risks, aside from conditions such as deep vein thrombosis, have not been thoroughly investigated. This study compares the in-hospital complication risks of TKA and UKA using a nationwide Japanese database.

**Methods:**

A retrospective cohort study was conducted using data from the Japanese Diagnosis Procedure Combination (DPC) database, spanning from April 2016 to March 2023. A total of 259,319 knee arthroplasty cases (TKA: 228,595; UKA: 30,724) were analyzed. Propensity score matching (1:1) was used to adjust for age, sex, comorbidities, and surgical factors, resulting in 30,591 matched pairs. Multivariable logistic regression analyses assessed the risks of complications, including deep vein thrombosis, pulmonary embolism, and surgical site infections.

**Results:**

Deep vein thrombosis is frequently observed as a complication with a high incidence rate. Even after propensity score matching, the incidence remained significantly higher in the TKA group (8.8%) compared with the UKA group (6.1%) (*p* < 0.0001). TKA was associated with significantly higher risks of deep vein thrombosis (odds ratio (OR): 1.467, 95% confidence interval (CI) 1.380–1.560, *p* < 0.0001), pulmonary embolism (OR: 1.709, 95% CI 1.182–2.470, *p* = 0.0044), and surgical site infection (OR: 1.512, 95% CI 1.277–1.790, *p* < 0.0001) compared with UKA. UKA showed lower risks of cognitive dysfunction, pneumonia, transfusion requirements, and shorter hospital stays. However, patients who underwent UKA had a higher risk of periprosthetic fractures.

**Conclusions:**

This study highlights the distinct risk profiles of TKA and UKA, emphasizing the need for tailored surgical decision-making. UKA offers advantages in reducing complications for specific patient populations. Strengthening prophylactic measures is crucial for effectively managing thromboembolic and infectious complications in patients undergoing TKA.

**Supplementary Information:**

The online version contains supplementary material available at 10.1186/s43019-025-00273-6.

## Introduction

Total knee arthroplasty (TKA) and unicompartmental knee arthroplasty (UKA) are important surgical options for managing osteoarthritis of the knee, each exerting distinct impacts on patient outcomes. As advancements in prosthesis development continue to evolve and mature, and the indications for UKA expand, its utilization has increased [1, 2]. UKA offers the advantage of preserving healthy parts of the knee joint, potentially leading to shorter recovery times and superior functional outcomes, particularly regarding range of motion and pain relief during recovery [3–5]. In contrast, while TKA typically requires a longer initial recovery period, it often provides more substantial long-term pain relief and improved joint function [6, 7].

Clinical outcomes of UKA have been reported to be comparable to or better than those of TKA, with fewer early postoperative complications [8–13], lower rates of early reoperation [9, 11–13], and reduced mortality [8, 9]. However, compared with TKA, UKA is associated with higher reoperation rates [9, 14]. The incidence of venous thromboembolism related to UKA is reported to be low, with symptomatic venous thromboembolism occurring in 0.41–1.6% of patients [15, 16], symptomatic deep vein thrombosis in 0.28–1.6% [15–19], and pulmonary embolism in 0.13% [15]. Several large consecutive series have reported no symptomatic venous thromboembolism [18] or asymptomatic venous thromboembolism following UKA [20]. Although systematic large-scale database studies on surgical site infections are limited, UKA has been reported to have a lower incidence of infections compared with TKA [21, 22]. Most studies have shown a tendency for reduced risk of complications such as deep vein thrombosis and pulmonary embolism following UKA compared with TKA. However, except for registry-based studies, the statistical power of these findings has often been insufficient owing to the small number of UKA cases and the lack of adjustment for confounding factors. Reports on complications such as deep vein thrombosis and pulmonary embolism are predominantly from Western countries, while studies on the incidence after lower limb surgery in Asian populations remain limited [21, 23, 24]. Furthermore, when assessing the incidence of venous thromboembolism, it is crucial to evaluate prophylactic antithrombotic therapy, as failing to do so may lead to incomplete assessments.

The Japanese Diagnosis Procedure Combination (DPC) database is a valuable resource for large-scale cohort studies in orthopedics, particularly for research on hip fractures [25–30]. However, the risk of complications associated with TKA and UKA in Japanese patients has not been thoroughly investigated. This study aims to compare the incidence of in-hospital complications between patients undergoing TKA and those undergoing UKA, utilizing a nationwide database of Japanese patients who underwent knee arthroplasty. To evaluate the complication risks of UKA relative to TKA, we conducted a large-scale nationwide case-cohort study using Japan’s insurance database. The study compared the incidence of complications, including deep vein thrombosis, pulmonary embolism, and surgical site infection, in cohorts matched for age, sex, body mass index, and comorbidities.

## Methods

### Study design

This retrospective study followed the ethical principles outlined in the Declaration of Helsinki. Data were retrospectively obtained from Japan’s nationwide administrative DPC reimbursement system database [31]. The present study utilized anonymized data from the DPC database, which is a nationwide administrative database used for medical reimbursement and healthcare quality assessment rather than a patient registry. The institutional review board approved the study, and an opt-out procedure was implemented to ensure transparency regarding data usage. In addition, this study does not include any information that could identify individual participants. The study period spanned from April 2016 to March 2023 and involved a nationwide survey targeting hospitals participating in Japan’s DPC system. During this period, approximately 1100 DPC-affiliated hospitals consistently submitted medical records and consented to their use for this research. Patients who underwent knee arthroplasty at these hospitals were included in the analysis, providing a comprehensive overview of the current state of knee arthroplasty in Japan. This clinical study analyzed patient groups who underwent TKA and UKA, focusing on postoperative complications in patients who underwent TKA and comparing them with those in patients who underwent UKA. Although UKA was previously considered unsuitable for patients with rheumatoid arthritis (RA), recent reports have demonstrated relatively favorable outcomes of UKA in patients with RA [32]. Therefore, patients with rheumatic diseases were also included in this study. Postoperative complications assessed in this study included pulmonary embolism, deep vein thrombosis, cerebrovascular disease, postoperative cognitive dysfunction, pneumonia, surgical site infection, and periprosthetic fracture. Postoperative cognitive dysfunction was identified using International Classification of Diseases-10th Revision (ICD-10) codes F010, F011, F012, F019, F03, F107, G238, G300, G301, G308, G309, G310, and G318, which encompass cognitive dysfunction and delirium occurring in the postoperative period, as described previously [27]. The primary diagnoses for knee replacement surgeries were classified on the basis of ICD-10 codes, with RA-assigned codes M0586, M0606, M0686, and M0696, and OA-assigned codes M170–M175 and M179. Patients in the TKA and UKA cohorts were identified using three key criteria: (1) primary diagnosis, (2) main reason for hospitalization, and (3) condition requiring the highest utilization of medical resources. Patients who underwent revision knee replacement surgery were excluded from this study.

### Propensity score matching

This study compared postoperative complications between patients who underwent TKA and those who underwent UKA. A 1:1 propensity score (PS) matching was performed to compare the two groups. This analysis adjusted for confounding factors, including age, sex, body mass index (BMI), and comorbidities such as hypertension, ischemic heart disease, cerebrovascular disease, chronic renal insufficiency, chronic pulmonary disease, diabetes, cognitive impairment, hyperlipidemia, and rheumatic disease. The discriminatory ability of the model was evaluated using the C-statistic. PS estimates were utilized to perform nearest-neighbor matching without replacement, applying a caliper set at 0.2 times the standard deviation of the PS estimates [30]. This approach generated matched pairs, forming TKA and UKA cohorts on the basis of PS matching.

### Statistical analyses

Data were expressed as mean ± standard deviation. Intergroup differences between patients who underwent TKA and those who underwent UKA were evaluated for each clinical parameter using the *χ*^2^ test and Student’s *t*-test. Differences in the use of antithrombotic agents between the TKA and UKA groups were also examined. After performing univariable logistic regression analysis for in-hospital complications, multivariable logistic regression analysis was conducted for severe in-hospital complications (such as deep vein thrombosis, pulmonary embolism, and surgical site infections) that were statistically significant. This analysis identified independent risk factors associated with these complications. Given the large sample size in this study, a stringent significance level was applied. All statistical tests were two-sided, and a *p*-value of less than 0.01 was considered statistically significant. Statistical analyses were performed using JMP version 17 (SAS, Cary, NC, USA).

## Results

**Fig. 1:**
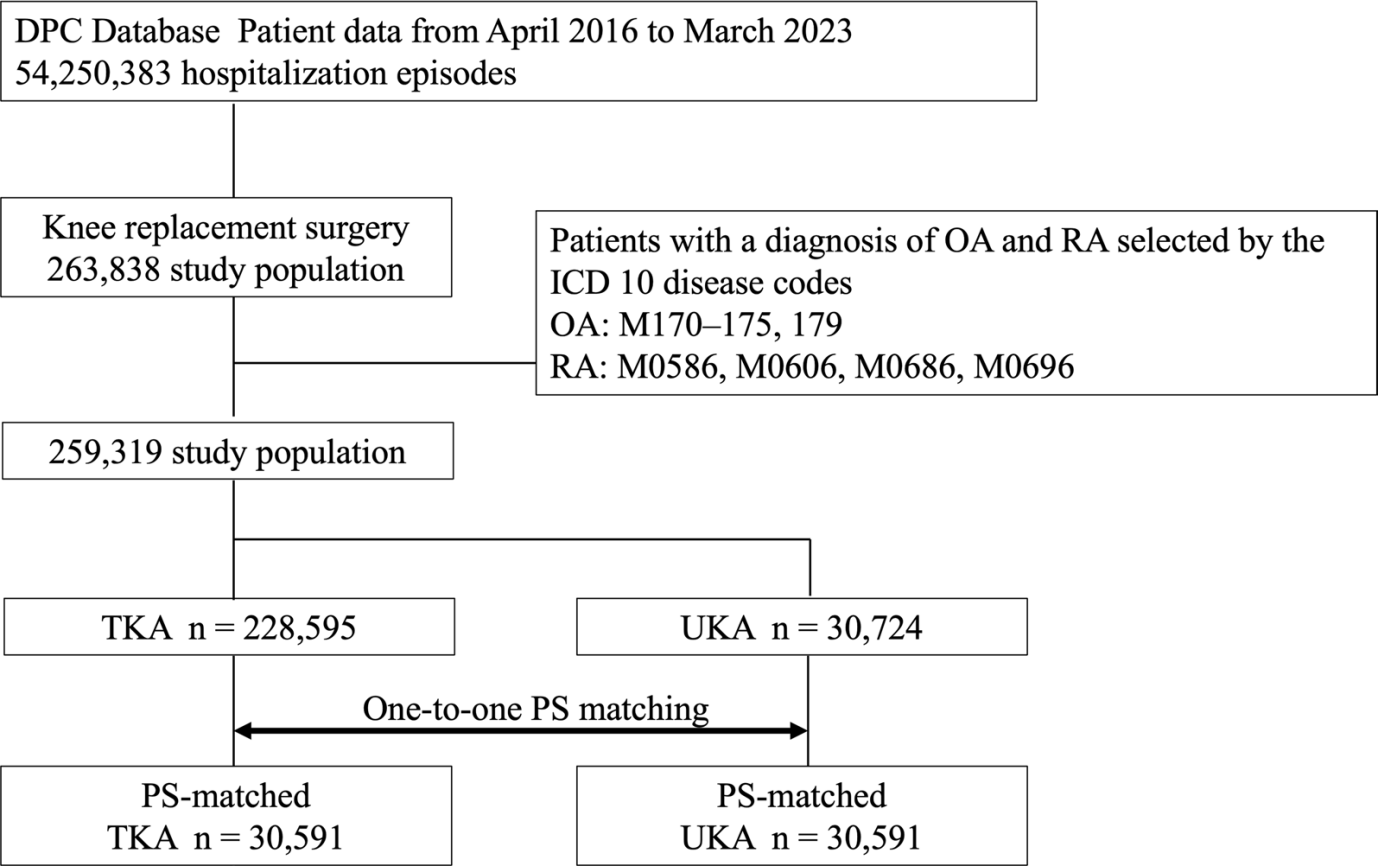
The flow diagram illustrates the patient selection process for total knee arthroplasty (TKA) and unicompartmental knee arthroplasty (UKA) patients who underwent knee replacement surgery due to osteoarthritis (OA) or rheumatoid arthritis (RA), as well as the propensity score (PS) matching process. It details the methodology used to extract target patients from the Diagnosis Procedure Combination (DPC) database and the subsequent PS matching process for the TKA and UKA cohorts

Figure 1 shows a schematic model of the patient selection process. From the dataset spanning from April 2016 to March 2023, a total of 259,319 patients who met the eligibility and exclusion criteria were identified. Among these, 228,595 patients underwent TKA, and 30,724 patients underwent UKA. After propensity score matching based on age, sex, BMI, the presence of simultaneous bilateral surgery, and comorbidities, both the TKA and UKA groups consisted of 30,591 cases each.

Table 1 summarizes the characteristics of patients who underwent TKA and UKA. Significant baseline differences were observed before PS matching, particularly in sex, BMI, comorbidities of rheumatic disease, and the frequency of simultaneous bilateral surgeries, with standardized mean differences (SMDs) of 0.1 or higher. The TKA group had a higher proportion of female patients, greater BMI, and a higher prevalence of rheumatic diseases, while the UKA group had a higher proportion of male patients. Simultaneous bilateral surgeries were performed in 6.7% of the TKA group and 4.2% of the UKA group.

**Table 1:** Characteristics of patients before and after propensity score matching

		Before PS matching			After PS matching			
Covariate	TKA	UKA	SMD	*p*-value	TKA	UKA	SMD	*p*-value
*n*	228,595	30,724			30,591	30,591		
Sex								
Men	47,839 (20.9%)	8487 (27.6%)	0.16	< 0.0001*	7932 (26.0%)	8459 (27.6%)	0.039	< 0.0001*
Women	180,756 (79.1%)	22,237 (72.4%)			22,659 (74.0%)	22,132 (72.4%)		
Age (years)	74.8 ± 7.8	74.3 ± 7.6	0.056	< 0.0001*	74.4 ± 7.7	74.3 ± 7.6	0.009	0.28
Body mass index	26.2 ± 4.9	25.3 ± 3.6	0.19	< 0.0001*	25.2 ± 3.8	25.3 ± 3.6	0.012	0.13
Hypertension	81,559 (35.7%)	9894 (32.2%)	0.073	< 0.0001*	9573 (31.3%)	9861 (32.2%)	0.02	0.012
Diabetes	49,373 (21.6%)	5801 (18.9%)	0.068	< 0.0001*	5420 (17.7%)	5783 (18.9%)	0.031	0.0001*
Cerebrovascular disease	7419 (3.3%)	879 (2.9%)	0.022	0.0003*	804 (2.6%)	877 (2.9%)	0.015	0.071
Ischemic heart disease	13,615 (6.0%)	1671 (5.4%)	0.022	0.0003*	1545 (5.0%)	1663 (5.4%)	0.017	0.032
Chronic renal dysfunction	6438 (2.8%)	698 (2.3%)	0.035	< 0.0001*	603 (2.0%)	696 (2.3%)	0.021	0.009*
Chronic lung disease	1350 (0.6%)	137 (0.5%)	0.02	0.0016*	117 (0.4%)	137 (0.4%)	0.01	0.21
Cognitive impairment	3594 (1.6%)	373 (1.2%)	0.031	< 0.0001*	345 (1.1%)	372 (1.2%)	0.008	0.31
Hyperlipidemia	45,250 (19.8%)	6087 (19.8%)	0.0004	0.94	5728 (18.7%)	6053 (19.8%)	0.027	0.0009*
Rheumatic disease	9105 (4.0%)	394 (1.3%)	0.18	< 0.0001*	423 (1.4%)	391 (1.3%)	0.009	0.26
Bilateral surgery	15,375 (6.7%)	1275 (4.2%)	0.11	< 0.0001*	1094 (3.6%)	1274 (4.2%)	0.03	0.0002*
Non-covariate	TKA	UKA	*F* value or *χ*^2^ statistics	*p*-value	TKA	UKA	*F* value or *χ*^2^ statistics	*p*-value
Charlson Comorbidity Index	0.70 ± 0.95	0.54 ± 0.85	850.7	< 0.0001*	0.61 ± 0.89	0.54 ± 0.85	101.7	< 0.0001*
Glucocorticoid use	6136 (2.7%)	341 (1.1%)	275.6	< 0.0001*	715 (2.3%)	340 (1.1%)	135.6	< 0.0001*
Length of hospitalization (days)	28.6 ± 15.9	22.9 ± 11.5	3666	< 0.0001*	28.3 ± 14.7	22.9 ± 11.5	2515	< 0.0001*
Mortality during hospitalization	104 (0.05%)	10 (0.03%)	1.0	0.31	15 (0.05%)	10 (0.03%)	1.0	0.32
Blood transfusion day 0 (unit)	0.06 ± 0.42	0.006 ± 0.12	531.0	< 0.0001*	0.05 ± 0.40	0.006 ± 0.12	425.5	< 0.0001*
Blood transfusion day 1 (unit)	0.04 ± 0.30	0.002 ± 0.07	395.6	< 0.0001*	0.03 ± 0.28	0.002 ± 0.07	332.7	< 0.0001*
Bone cement use	206,532 (90.4%)	29,003 (94.4%)	533.3	< 0.0001*	27,721 (90.6%)	28,874 (94.4%)	313.3	< 0.0001*
Navigation use	65,889 (28.8%)	8096 (26.4%)	81.2	< 0.0001*	8807 (28.8%)	8058 (26.3%)	45.9	< 0.0001*

One-to-one PS matching was performed

Data are shown as mean ± standard deviation; *p-values of < 0.01 are considered significant by the Student’s *t*-test and *χ*^2^ test difference

*TKA* total knee arthroplasty, *UKA* unicompartmental knee arthroplasty

After 1:1 propensity score matching, the SMDs for all variables were adjusted to less than 0.1, indicating that differences in age, sex, BMI, comorbidities, and surgical type between the TKA and UKA groups were appropriately balanced. However, the Charlson Comorbidity Index remained higher in the TKA group. When the continued use of oral glucocorticoids at a dose of 5 mg or more was defined as glucocorticoid use, the TKA group had a higher prevalence of glucocorticoid users. In addition, hospital stay was longer, and the rate of blood transfusion on the day of surgery and the following day was higher in the TKA group. The in-hospital mortality rate showed no difference between the two groups. Cemented knee arthroplasty was predominant (> 90%) in both groups, with an even higher proportion in the UKA group. The utilization of navigation systems was less than 30% in both groups but higher in the OA group. The C-statistic was calculated at 0.726.

Table 2 summarizes the use of anticoagulants and antiplatelet agents for thromboprophylaxis. Edoxaban was the most frequently used medication in both groups. Before PS matching, significant differences were observed in all medications except clopidogrel. Edoxaban, enoxaparin, warfarin, and apixaban were more commonly used in the TKA group, while fondaparinux and aspirin were more frequently used in the UKA group. After PS matching, the differences in antithrombotic medication use remained consistent. Although thromboprophylaxis was more prevalent in the TKA group, appropriate use of antithrombotic agents was also observed in the UKA group.

**Table 2:** Comparison of antithrombotic therapies before and after propensity score matching

	Before PS matching				After PS matching			
	TKA	UKA	*χ*^2^ statistics	*p*-value	TKA	UKA	*χ*^2^ statistics	*p*-value
Edoxaban	141,436 (61.9%)	15,933 (51.9%)	1138	< 0.0001*	19,055 (62.3%)	15,874 (51.9%)	675.1	< 0.0001*
Fondaparinux	4526 (2.0%)	860 (2.8%)	89.4	< 0.0001*	575 (1.9%)	859 (2.8%)	57.6	< 0.0001*
Enoxaparin	22,598 (9.9%)	2469 (8.0%)	106.1	< 0.0001*	3024 (9.9%)	2454 (8.0%)	65.1	< 0.0001*
Aspirin	18,959 (8.3%)	3122 (10.2%)	121.3	< 0.0001*	2316 (7.1%)	3098 (10.1%)	123.9	< 0.0001*
Warfarin	5121 (2.2%)	342 (1.1%)	166.8	< 0.0001*	658 (2.2%)	340 (1.1%)	103.0	< 0.0001*
Clopidogrel	6313 (2.8%)	838 (2.7%)	0.1	0.73	782 (2.6%)	833 (2.7%)	1.7	0.20
Apixaban	5913 (2.6%)	526 (1.7%)	85.6	< 0.0001*	807 (2.6%)	522 (2.2%)	62.5	< 0.0001*

One-to-one PS matching was performed

*P*-values of < 0.01 are considered significant by the *χ*^2^ test; *PS* propensity score

Table 3 presents the analysis comparing the incidence of complications between the TKA and UKA groups. Before PS matching, the TKA group had significantly higher risks of deep vein thrombosis, pulmonary embolism, postoperative cerebrovascular events, postoperative cognitive dysfunction, pneumonia, and surgical site infections, whereas periprosthetic fractures were significantly more common in the UKA group. After PS matching, the same trend persisted, with the higher incidence of deep vein thrombosis and surgical site infections in the TKA group remaining robust.

**Table 3:** Comparison of complications before and after propensity score matching

	Before PS matching				After PS matching			
	TKA	UKA	*χ*^2^ statistics	*p*-value	TKA	UKA	*χ*^2^ statistics	*p*-value
Deep vein thrombosis	21,262 (9.3%)	1882 (6.1%)	336.1	< 0.0001*	2681 (8.8%)	1876 (6.1%)	153.6	< 0.0001*
Pulmonary embolism	686 (0.3%)	45 (0.2%)	22.7	< 0.0001*	77 (0.3%)	45 (0.3%)	8.4	0.0037*
Cerebrovascular disorder	812 (0.4%)	74 (0.2%)	10.4	0.001*	104 (0.3%)	74 (0.2%)	5.1	0.024
Cognitive dysfunction	1713 (0.8%)	123 (0.4%)	46.9	< 0.0001*	221 (0.7%)	122 (0.4%)	28.7	< 0.0001*
Pneumonia	444 (0.2%)	28 (0.1%)	15.8	< 0.0001*	54 (0.2%)	27 (0.1%)	9.0	0.0027
Surgical site infection	2739 (1.2%)	228 (0.7%)	49.8	< 0.0001*	340 (1.1%)	228 (0.7%)	22.3	< 0.0001*
Periprosthetic fracture	139 (0.1%)	47 (0.2%)	32.1	< 0.0001*	19 (0.1%)	47 (0.2%)	11.9	0.0006*

One-to-one PS matching was performed

*P-values of < 0.01 are considered significant by the *χ*^2^ test; *PS* propensity score

Table 4 presents the univariable logistic regression analysis results, examining the association between TKA and postoperative complications. TKA was identified as a significant risk factor for several complications, with an odds ratio of 1.471 (95% CI 1.383–1.563, *p* < 0.0001) for deep vein thrombosis, 1.713 (95% CI 1.185–2.475, *p* = 0.0042) for pulmonary embolism, and 1.497 (95% CI 1.264–1.771, *p* < 0.0001) for surgical site infections. Pneumonia and postoperative cognitive dysfunction were also significantly associated with TKA. Conversely, the risk of periprosthetic fractures was lower in TKA, with an odds ratio of 0.404 (95% CI 0.237–0.688, *p* = 0.0009).

**Table 4:** Association between occurrence of complications and total knee arthroplasty

	Total (n)	TKA (n)	Odds Ratio (95% CI)	χ^2^ statistics	*p*-value
Deep vein thrombosis	4557	2681	1.471 (1.383–1.563)	154.4	< 0.0001*
Pulmonary embolism	122	77	1.713 (1.185–2.475)	8.5	0.0042*
Cerebrovascular disorder	178	104	1.407 (1.044–1.896)	5.1	0.025
Cognitive dysfunction	343	221	1.817 (1.456–2.268)	29.1	< 0.0001*
Pneumonia	81	54	2.001 (1.261–3.178)	9.2	0.0033*
Surgical site infection	568	340	1.497 (1.264–1.771)	22.4	< 0.0001*
Periprosthetic fracture	66	19	0.404 (0.237–0.688)	12.3	0.0009*

^*^P-values of < 0.01 are considered significant by the *χ*^2^ test; *TKA* total knee replacement, *CI* confidence interval

**Fig. 2:**
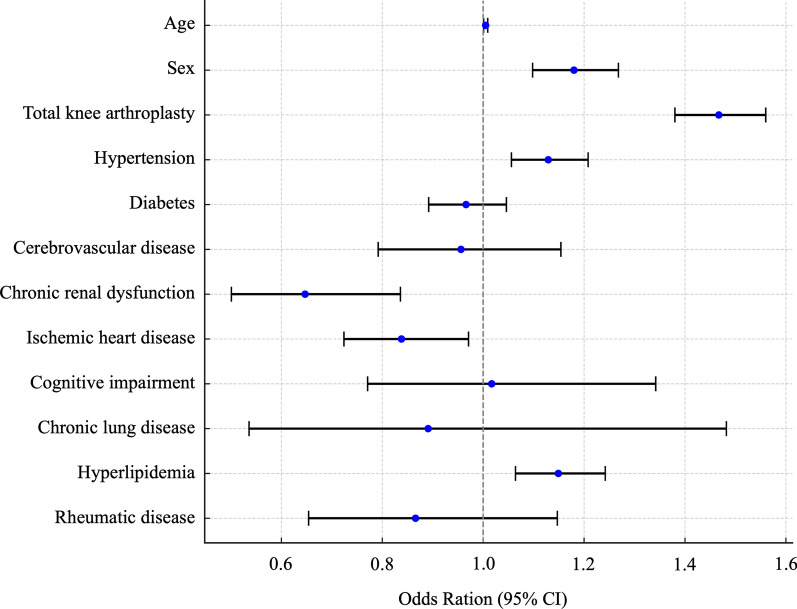
Multivariate analysis of risk factors for deep vein thrombosis after knee replacement. The forest plot shows odds ratios (ORs) with 95% confidence intervals (CIs). The reference line at OR = 1 indicates no effect

Table 5 presents the multivariable logistic regression analysis results for factors associated with deep vein thrombosis in hospitalized patients undergoing knee arthroplasty. Among the variables examined, female sex, TKA, hypertension, and hyperlipidemia emerged as significant independent risk factors. TKA was identified as a major risk factor, with an odds ratio of 1.467 (95% CI 1.380–1.560, *p* < 0.0001). Age and other comorbidities were not significant risk factors. Figure 2 shows a forest plot illustrating the results of a multivariate logistic regression analysis of risk factors for deep vein thrombosis.

**Table 5:** Multivariate logistic analysis for risk factors for deep vein thrombosis after knee replacement surgery during hospitalization

Variable	Odds ratio (95% CI)	*χ*^2^ statistics	*p*-value
Age	1.005 (1.001–1.009)	5.7	0.017
Sex (female)	1.180 (1.098–1.268)	20.7	< 0.0001*
Total knee arthroplasty	1.467 (1.380–1.560)	152.5	< 0.0001*
Hypertension	1.129 (1.056–1.208)	12.5	0.0004*
Diabetes	0.966 (0.892–1.046)	0.7	0.39
Cerebrovascular disease	0.956 (0.792–1.154)	0.2	0.64
Chronic renal dysfunction	0.647 (0.501–0.836)	12.6	0.0009*
Ischemic heart disease	0.838 (0.724–0.971)	5.8	0.018
Cognitive impairment	1.017 (0.771–1.342)	0.01	0.9
Chronic lung disease	0.891 (0.536–1.482)	0.2	0.66
Hyperlipidemia	1.149 (1.064–1.242)	12.3	0.0004*
Rheumatic disease	0.866 (0.654–1.147)	1.0	0.32

^*^P-values of < 0.01 are considered significant by the *χ*^2^ test; *CI* confidence interval

**Fig. 3:**
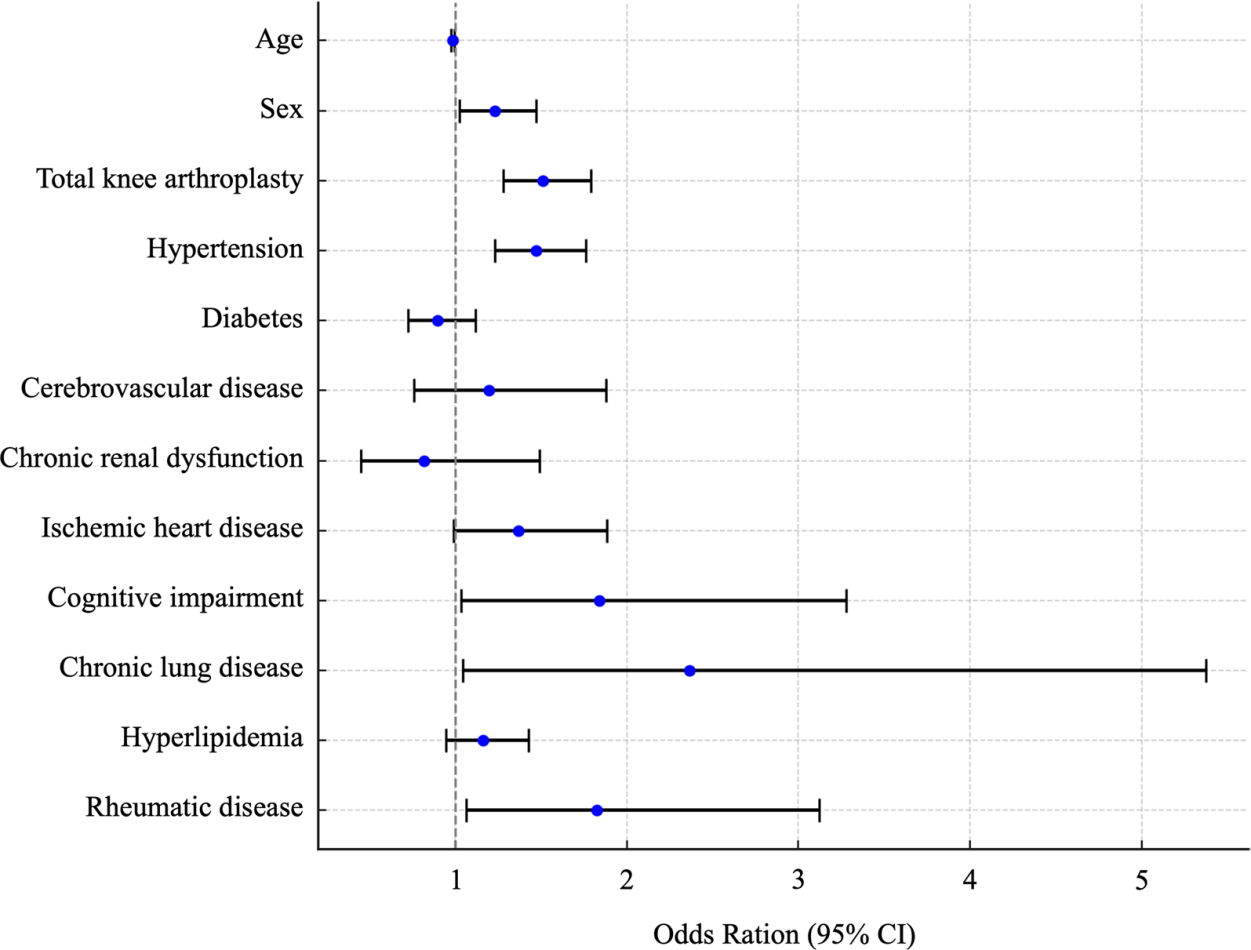
Multivariate analysis of risk factors for surgical site infection after knee replacement. The forest plot shows odds ratios (ORs) with 95% confidence intervals (CIs). The reference line at OR = 1 indicates no effect

Table 6 presents the multivariable logistic regression analysis results for factors associated with surgical site infections in hospitalized patients undergoing knee arthroplasty. TKA and hypertension were identified as significant independent risk factors. TKA was the primary risk factor, with an odds ratio of 1.512 (95% CI 1.277–1.790, *p* < 0.0001), and hypertension also increased SSI risk, with an odds ratio of 1.471 (95% CI 1.231–1.758, *p* < 0.0001). In contrast, age, sex, and other comorbidities were not found to be significant risk factors. Figure 3 shows a forest plot illustrating the results of a multivariate logistic regression analysis of risk factors for surgical site infection.

**Table 6:** Multivariate logistic analysis for risk factors for surgical site infection after knee replacement surgery during hospitalization

Variable	Odds ratio (95% CI)	*χ*^2^ statistics	*p*-value
Age	0.983 (0.972–0.993)	9.9	0.0016*
Sex (male)	1.228 (1.024–1.472)	4.8	0.026
Total knee arthroplasty	1.512 (1.277–1.790)	23.6	< 0.0001*
Hypertension	1.471 (1.231–1.758)	17.6	< 0.0001*
Diabetes	0.898 (0.722–1.118)	0.9	0.34
Cerebrovascular disease	1.193 (0.758–1.877)	0.6	0.44
Chronic renal dysfunction	0.816 (0.447–1.491)	0.5	0.51
Ischemic heart disease	1.366 (0.991–1.884)	3.3	0.057
Cognitive impairment	1.838 (1.031–3.279)	3.6	0.039
Chronic lung disease	2.366 (1.042–5.374)	3.3	0.04
Hyperlipidemia	1.160 (0.945–1.425)	2.0	0.16
Rheumatic disease	1.822 (1.064–3.121)	4.0	0.029

^*^p-values of < 0.01 are considered significant by the *χ*^2^ test; *CI* confidence interval

Supplemental Table 1 presents the multivariable logistic regression analysis results for factors associated with postoperative pulmonary embolism in patients who underwent knee arthroplasty. TKA was the only significant independent risk factor, with an odds ratio of 1.709 (95% CI 1.182–2.470, *p* = 0.0044).

## Discussion

The results of this study revealed that TKA was associated with significantly higher risks of deep vein thrombosis with an odds ratio of 1.467 (95% CI 1.380–1.560, *p* < 0.0001), pulmonary embolism with an odds ratio of 1.709 (95% CI 1.182–2.470, *p* = 0.0044), and surgical site infections with an odds ratio of 1.512 (95% CI 1.277–1.790, *p* < 0.0001) compared with UKA. Conversely, UKA was associated with a higher risk of periprosthetic fractures. These findings were derived from a nationwide analysis using the Japanese DPC database, which allowed for a large-scale comparison of postoperative complications between TKA and UKA.

The comparison between the results of the current study and previous research is presented in Supplemental Table 2 [13, 20, 33–36]. The lower venous thromboembolism risk in UKA compared with TKA observed in this study aligns with previous reports. Lombardi et al. reported no symptomatic venous thromboembolism events in 423 consecutive patients who underwent UKA [20]. In a large-scale study, Hansen et al. found significantly lower deep vein thrombosis and pulmonary embolism risks in patients who underwent UKA using propensity score matching [13]. Similarly, Brown et al. observed a lower venous thromboembolism incidence in UKA (0.64% versus 1%) across 2840 cases, although this difference was not statistically significant [33]. These findings support the expected advantage of UKA in reducing thromboembolic risks.

The increased thromboembolic risk in the TKA group may be attributed to several factors. TKA generally requires a longer operative time than UKA, leading to prolonged surgical stress and a greater risk of postoperative immobility, which may contribute to deep vein thrombosis and pulmonary embolism [37, 38]. A longer operative time has also been reported to be associated with surgical site infections. Therefore, the longer operative time in the TKA group compared with the UKA group may have contributed to the increased risk of surgical site infections. Studies have shown that surgeries exceeding 100 min are associated with a higher incidence of deep infections [39]. Although patient backgrounds were matched through propensity score matching, differences in corticosteroid use may have influenced the results. Glucocorticoids are known to alter coagulation pathways and may contribute to an increased risk of thromboembolic events [40]. As this study did not assess operative time, investigating the association between operative time and complications in large-scale database studies remains an important subject for future research. A recent national database study from Germany also reported that the 1-year incidence of periprosthetic joint infection was 0.5% for UKA and 2.8% for TKA, consistent with our findings that UKA has a lower risk of infection [34]. A larger implant surface area potentially provides a favorable environment for bacterial colonization, increasing the risk of periprosthetic joint infection [41]. The findings of this study align with previous research while also providing novel insights.

In Japan, as shown in this study, edoxaban is widely used to prevent thromboembolism. Mechanical methods such as elastic stockings and foot pumps are also commonly utilized [42]. Internationally, low-molecular-weight heparin (LMWH), direct oral anticoagulants (DOACs) such as apixaban and rivaroxaban, and aspirin are commonly used for deep vein thrombosis (DVT) prevention [43]. There do not appear to be any reported significant differences in infection control measures between Japan and other countries. Standard measures include perioperative antibiotic administration, sterile surgical techniques, and postoperative wound care [44].

This study is a large-scale database analysis comparing 30,591 propensity-matched pairs of patients who underwent TKA and UKA with matched patient backgrounds, including age, sex, body mass index, and various comorbidities. It also provides a detailed evaluation of antithrombotic therapies used for the prevention of deep vein thrombosis and pulmonary embolism. The balanced cohort and comprehensive thromboembolic risk assessment under adequate thromboprophylaxis offer valuable data complementing existing evidence. Moreover, as evidence on pulmonary embolism risk differences between TKA and UKA remains limited, the findings of this study have the potential to serve as a critical reference for knee surgeons in making informed surgical decisions.

With its lower complication rates for specific adverse events, UKA may be a safer option for the elderly or certain patient populations. This study demonstrated that UKA is associated with lower risks of postoperative cognitive dysfunction and pneumonia compared with TKA, which could facilitate more efficient rehabilitation and potentially broaden the indications for UKA in clinical practice. However, in cases of severe joint deformity, TKA is indicated instead of UKA [45]. The increased risks associated with TKA, such as deep vein thrombosis, pulmonary embolism, and surgical site infections, necessitate enhanced preventive measures. These include optimized antithrombotic therapy and stricter infection control protocols.

It is important to note that reports indicate inferior long-term outcomes for UKA in patients with severe obesity [46, 47], necessitating careful decision-making when selecting the surgical method. Reports suggest that UKA in patients under 55 years of age tends to have a higher risk of revision than those aged 55–64 years, with revision risks reported as 19% versus 12% [48]. However, a meta-analysis has shown that the revision risk for UKA in younger patients is not significantly higher [49], indicating the need for further validation in future studies. UKA is not always an alternative to TKA, as it is only suitable for patients with isolated unicompartmental knee osteoarthritis and is contraindicated in conditions such as tricompartmental osteoarthritis or active inflammatory arthritis. Therefore, surgical decision-making must be tailored to individual patient pathology. Given these considerations, careful decision-making is crucial when selecting the surgical method. A strategy emphasizing appropriate patient selection for UKA could improve outcomes and reduce healthcare costs, underscoring the importance of tailored approaches in knee arthroplasty.

The major strength of this study lies in its use of a nationwide database and the application of propensity score matching to adjust for confounding factors such as age, sex, body mass index, and comorbidities. Furthermore, the large sample size enhances the statistical reliability of the results. However, there are several limitations to this large study, as outlined below. First, the study population was limited to patients who underwent knee replacement surgery in acute care hospitals included in the DPC data system. This excludes patients admitted to non-DPC-reported beds, which account for approximately 30% of all general hospital beds, and patients never treated in an acute care hospital [25]. Second, this study is limited by the inability to verify the accuracy of DPC disease classifications and assess the severity of symptoms associated with comorbidities in the actual patients. Certain aspects of the diagnostic methods for complications remain unverified. For instance, the assessment of deep vein thrombosis, including imaging modalities such as computed tomography and ultrasound, timing of diagnosis, anatomical location, and severity, has not been comprehensively investigated. Third, this study is limited by the inability to confirm the severity of knee joint deformities and the specifics of the surgical approach or equipment used. Fourth, the inability to assess factors affecting transfusion rates, such as the use of tranexamic acid, and the lack of a standardized method for evaluating venous thromboembolism risk, such as the Caprini score, are also limitations of this study. Lastly, long-term outcomes such as infection, periprosthetic fracture, reoperation, and mortality after discharge were not evaluated. Further large-scale studies utilizing detailed patient data are necessary to address these limitations.

## Conclusions

Evaluating postoperative complication risks for TKA and UKA under rigorous PS matching provides valuable insights for optimizing surgical decision-making tailored to individual patients. Compared with TKA, UKA demonstrated reduced risks of deep vein thrombosis, pulmonary embolism, surgical site infections, decreased transfusion requirements, and shorter hospital stays. However, the increased risk of postoperative fractures with UKA warrants careful consideration.

These findings suggest that thromboprophylaxis strategies should be tailored on the basis of surgical procedures, with more aggressive anticoagulation considered for TKA to mitigate its higher thromboembolic risk. In addition, enhanced perioperative infection control measures, particularly for TKA, could help reduce surgical site infections.

This study reflects the current state of knee arthroplasty in Japan and highlights key areas for improving patient outcomes. Future research should focus on optimizing individualized perioperative management, including thromboprophylaxis, infection prevention, and rehabilitation strategies, to improve knee arthroplasty procedures’ safety and efficacy.

## Supplementary Information

Additional File 1.

## Data Availability

The data that support the findings of this study are available upon request from the corresponding author.

## Abbreviations

CI: Confidence interval
DPC: Diagnosis procedure combination
OA: Osteoarthritis
PS: Propensity score
RA: Rheumatoid arthritis
TKA: Total knee arthroplasty
UKA: Unicompartmental knee arthroplasty
