# Supplementary material for: Thromboembolic and infectious complication risks in TKA and UKA: evidence from a Japanese nationwide cohort
Source: Knee Surg Relat Res. 2025 May 8;37:19. doi: 10.1186/s43019-025-00273-6 (PMC12063263; doi:10.1186/s43019-025-00273-6)
Supplement: Supplementary file 1 — Additional File 1. [file 43019_2025_273_MOESM1_ESM.docx]

Supplemental Table 1 Multivariate logistic analysis for risk factors for pulmonary embolism after knee replacement surgery during hospitalization

| Variable | Odds Ratio (95% CI) | χ2 statics | *p*-value |
| --- | --- | --- | --- |
| Age | 1.021 (0.995-1.046) | 2.7 | 0.1 |
| Sex (Female) | 1.289 (0.836-1.988) | 1.4 | 0.25 |
| Total knee arthroplasty | 1.709 (1.182-2.470) | 8.4 | 0.0044* |
| Hypertension | 1.203 (0.815-1.775) | 0.9 | 0.35 |
| Diabetes | 0.986 (0.620-1.569) | 0.003 | 0.95 |
| Cerebrovascular disease | 1.122 (0.410-3.067) | 0.05 | 0.82 |
| Chronic renal dysfunction | 0.374 (0.052-2.686) | 1.3 | 0.33 |
| Ischemic heart disease | 1.569 (0.813-3.028) | 1.6 | 0.18 |
| Cognitive impairment | 1.325 (0.326-5.384) | 0.14 | 0.69 |
| Chronic lung disease | 2.086 (0.288-15.11) | 0.42 | 0.47 |
| Hyperlipidemia | 1.018 (0.645-1.607) | 0.006 | 0.94 |
| Rheumatic disease | 0.611 (0.085-4.389) | 0.28 | 0.62 |

**p*-values of < 0.01 are considered significant by the χ2 test; CI means confidence interval.

Supplemental Table 2 Comparison of Deep Vein Thrombosis, Pulmonary Embolism, and Surgical Site Infection Risks in TKA and UKA Across Studies

| Study | Sample Size | DVT Risk (%) | PE Risk (%) | SSI Risk (%) | Notes |
| --- | --- | --- | --- | --- | --- |
| Current Study | TKA (n=30,591) vs. UKA (n=30,591) | TKA > UKA (OR: 1.47, 95% CI: 1.38-1.56) | TKA > UKA (OR: 1.71, 95% CI: 1.18-2.47) | TKA > UKA (OR: 1.51, 95% CI: 1.28-1.79) | Japanese DPC database analysis |
| Lombardi et al. (2007) ref 20 | UKA (n=423) | 0% | 0% | Not reported | No symptomatic thromboembolic events observed |
| Hansen et al. (2019) ref 13 | Large US database study | TKA > UKA (OR: 1.58, 95% CI: 1.48-1.70) | TKA > UKA (OR: 1.51, 95% CI: 1.26-1.81) | TKA > UKA (OR: 1.32, 95% CI: 1.24-1.40) | Propensity score matching analysis |
| Brown et al. (2012) ref 33 | TKA (n=2,840) vs. UKA (n=2,840) | 1.0% vs. 0.64% | Not reported | 1.64% vs. 0.71% | Multicenter analysis, higher morbidity in TKA |
| Szymski et al. (2024) ref 34 | TKA (n=264,137) vs. UKA (n=36,861) | Not reported | Not reported | 0.5% vs. 2.8% | One-year postoperative infection rate |
| Burnett III et al. (2021) ref 35 | TKA (n=2,383) vs. UKA (n=2,383) | 5.0% vs. 3.1% | 1.5% vs. 0.8% | Not reported | Matched cohort study over ten years |
| Burn et al. (2019) ref 36 | TKA (n=250,377) vs. UKA (n=32,379) | 1.00 vs. 0.62 | Not reported | 1.00 vs. 0.85 | Population-based network study |

TKA means total knee arthroplasty; UKA means unicompartmental knee arthroplasty; DVT means deep vein thrombosis; PE means pulmonary embolism; SSI means surgical site infection; OR means odds ratio; CI means confidential interval.
